# Speech of Lieut.-Gen. Sir Howard Douglas, Bart. M.P. on the Army Surgeon's Bill

**Published:** 1847-07-01

**Authors:** 


### Speech of Lieut.-General Sir Howard Douglas, Bart., M.P. for Liverpool, on the Army Surgeons' Bill, in the House of Commons, Monday, April 12, 1847.

We publish with real satisfaction certain passages in the speech of the gallant Sir Howard Douglas, on the ill-treatment of the Army Surgeons—a class of officers forming a considerable portion of the entire body of the medical profession, whose honour and reputation it has nobly upheld, under every circumstance of service—in peace and in war.

The friends of the Army Surgeons, *in the Army,* have at all times been of the class to which we know that Sir Howard Douglas belongs—those emphatically designated as of "*the heroic school*"—the school of Abercrombie, Moore, Hope, and Graham.

The honoured commanders named were sterling friends to our brethren in the army; but the two first were killed in action ere they had done more than record their sentiments on the merits and deserts of the surgeons whose conduct in battle had excited their admiration and gratitude. The others, owing to party or political circumstances, were impeded in their endeavours. Sir Howard Douglas, then, follows in a noble wake.

After exposing the low rate of pay comparatively, causing surgeons "to cling to active service long after their physical powers were too much impaired to discharge sufficiently their laborious duties," the gallant General proceeded in detail to recite the grievances of "that learned, most important, and, as he thought, rather neglected class, entitled on every account to the first consideration and distinction."

"The army surgeon, it is true, does not purchase his commission; but the expense necessarily invested on his education must not be forgotten; and he, like the paymaster, cannot realize this by sale, for the benefit of his family. No one who knows anything of the severe and painful duties which a medical officer has to discharge, and the services in which he must be engaged, in the presence of disease of every kind, facing death in every shape, can doubt of the hardships which officers of that class must have undergone during a service of thirty years. The surgeon, too, let it be remembered, must be in full possession of all his energies, keep himself well up to the mark in every improvement in practical surgery and in medical science. Paymasters and quartermasters may, with rather diminished powers, cling to the service without any great detriment to it, or inconvenience to themselves, after these had somewhat faded; but there was a period of life beyond which the medical officer, however perfect his intellect, could not discharge effectually his duties on the field as surgeon of the regiment—a period beyond which vision becomes imperfect, the nerve unbraced, the hand too unsteady, in the difficult and delicate operations which a surgeon was called upon to perform. No man of the age of sixty or sixty-two could be expected to retain these faculties unimpaired; and as the great bulk of medical officers enter the army at the age of twenty-four, thirty-two years' service would bring them near to that stage in human existence, beyond which it would be vain and unreasonable to expect that a medical officer of thirty or thirty-five years service in all climates should retain his efficiency. Officers so circumstanced are aware of this sad truth, but continue to serve, knowing that the half-pay to which they are entitled is insufficient for their wants; that, from bodily infirmities and deficiency of mental energy, they cannot add to their income by private practice, being unequal to compete successfully with younger and more recently educated practitioners, and therefore cling to the service. It is for the interests of the service, then, that men so circumstanced should cede their avocations to younger individuals; but this they can only be tempted to do, or are justified in doing, by our increasing their rates of retirement. The medical officer, while on full-pay, has no cause for complaint—this is sufficient to attach him to the service, but binds him too long, from the rates of retirement being so inadequate. The hon. and gallant Member then cited, from official returns, the cases of many deputy inspectors general of hospitals, and surgeons, who had been from thirty-six to forty-three years in the service.

"Having explained the ordinary duties of medical officers, he would now beg to say a few words which he hoped would take these officers as a class out of the category of civil functionaries and non-combatants. The medical officer cannot be considered a non-combatant in the sense of personal exposure, nor in many cases, as he could tell, of personal military gallantry. The medical officer is suddenly called upon to discharge duties which require the vigour and powers of endurance of less advanced age; to partake of the fatigue, privations, and diseases incidental to actual service; to brave every climate, to witness death in every form, and to suffer it himself in the field, when administering to those whose lives he is endeavouring to preserve. Two surgeons of Her Majesty's regiments were killed on the field of battle in the operations of the Sutlez; three at Cabul. How many of the Honourable Company's service he knew not. He requested the House to let him endeavour to depict the duties of medical officers on the field of battle. The action is about to commence. The medical officers attached to the troops take post in the immediate rear of their respective corps, and then prepare the implements of their mournful and painful calling. The battle begins; the active combatants, unmindful and regardless of danger, buoyed above the terror of death or of wounds by ardour and excitement, which few can imagine, heed not the casualties that happen around them. Not so the medical officer. The fallen and the disabled that are not beyond the reach of his skill, become the subjects of his immediate care. The fiercer the fight, the more numerous these sad consignments. There, on the naked field, exposed to personal risk, and within reach of the bullets, which may have previously ploughed the ranks of the columns, or lines in his front, the medical officers, with unflinching eye, steady hand, and well-braced nerves, discharge their melancholy functions, and frequently lose their lives in endeavouring to save others. Is the battle won? The troops move forward with exultation to reap the fruits of their victory. The medical officers remain on the blood-stained field, amidst the havoc of war, to collect the mutilated victims, and administer to the sacrifices that victory exacts. Is the battle lost? or is the field, though won, abandoned, as ofttimes happens? The medical officers perform their still more painful duties on the forsaken field, and become themselves captives, in common with those who, by their aid, may survive. Then there was the assault of the fortress, and the storming of the breach, at which medical officers are invariably aiding. And this is the class—such the persons, from whom you withhold advantages enjoyed by the practitioners of a less exalted surgery. The horse is a noble animal; the veterinary science is an important and useful profession; but man is a nobler animal still, and a soldier, apart from other considerations of humanity, a more important and valuable agent. He appealed, then, in the strongest terms, to the feelings and generosity of Her Majesty's Government, to the House, and to the country, against the exclusion of the class of officers whose case he had taken up, from advantages which are extended to others—from a boon which all public servants, civil and military, now enjoy. The hon. and gallant member stated that he would not enter further into detail, but leave the case to the consideration of the House and the Government, and he hoped he had not appealed in vain to the noble Lord at the head of the Government, and would be consoled by the reflection that he had done his best in behalf of these officers."

